# Gold-thickness-dependent Schottky barrier height for charge transfer in metal-assisted chemical etching of silicon

**DOI:** 10.1186/1556-276X-8-193

**Published:** 2013-04-26

**Authors:** Zewen Zuo, Guanglei Cui, Yi Shi, Yousong Liu, Guangbin Ji

**Affiliations:** 1 College of Physics and Electronics Information, Anhui Normal University, Wuhu 241000, China; 2 School of Electronic Science and Engineering, Nanjing University, Nanjing 210093, China; 3 College of Materials Science and Technology, Nanjing University of Aeronautics and Astronautics, Nanjing 211100, China

**Keywords:** Silicon nanowires, Etching rate, Schottky barrier height, Thickness dependent

## Abstract

Large-area, vertically aligned silicon nanowires with a uniform diameter along the height direction were fabricated by combining *in situ*-formed anodic aluminum oxide template and metal-assisted chemical etching. The etching rate of the Si catalyzed using a thick Au mesh is much faster than that catalyzed using a thin one, which is suggested to be induced by the charge transport process. The thick Au mesh in contact with the Si produces a low Au/Si Schottky barrier height, facilitating the injection of electronic holes from the Au to the Si, thus resulting in a high etching rate.

## Background

Silicon nanowires (SiNWs) have attracted significant research interest because of their unique properties and potential applications as building blocks for advanced electronic devices [1,2], biological and chemical sensors [2-4], and optoelectronic devices [5] as well as photovoltaic devices [2,6,7]. Metal-assisted chemical etching has attracted increasing attention in the recent years because of its simplicity and low cost coupled with its excellent control ability on the structural and electrical parameters of the resulting SiNWs [8-13]. In metal-assisted chemical etching, the formation rate of SiNWs, i.e., the etching rate of Si substrate, is controlled by the mass transfer process of the reagent, including the by-product, and by the charge transfer process during the Si etching [13,14].

The crystallographic orientation and the doping properties of the Si substrate, the type and the structure of a noble metal, the component and the concentration of the etching solution, temperature, illumination, and so on were reported to have a substantial effect on the etching rate [11,12,14-17]. In the present study, the thickness of the Au catalyst film, which is a new control dimension, was found to affect the etching rate of Si during the fabrication of SiNWs by a method that combines the anodic aluminum oxide (AAO) template and the metal-assisted chemical etching. The aforementioned method results in the formation of large-area, vertically aligned SiNW arrays with a uniform diameter along the height direction. Furthermore, the method shows better control on the diameter, spacing, and density of SiNW arrays.

## Methods

**Figure 1:**
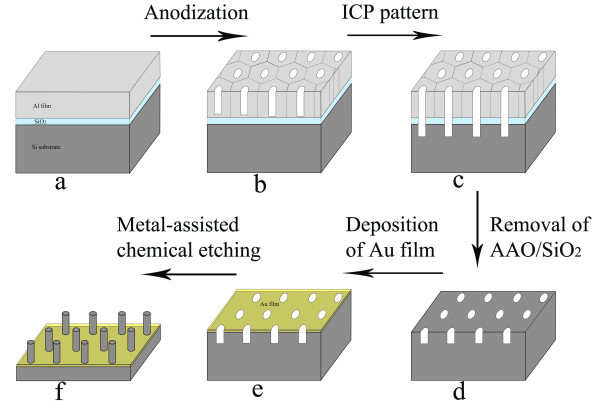
**Schematic of the SiNW fabrication process.** (**a**) Depositing an Al film on the SiO2/Si substrate. (**b**) Anodization of the Al film to form AAO mask. (**c**) Excavating the barrier layer and SiO2 layer as well as patterning the Si surface by ICP etching. (**d**) Removal of the AAO/SiO2 layer to achieve patterned Si substrate. (**e**) Depositing a Au film on patterned Si substrate. (**f**) Metal-assisted chemical etching to obtain Si nanowire array.

Figure 1 schematically illustrates the basic experimental procedure employed in this study. First, a 50-nm-thick SiO_2_ film was deposited by plasma-enhanced chemical vapor deposition on a (100)-oriented silicon substrate (p-type, 1 to 10 Ω cm), which was precleaned by a standard RCA procedure. Subsequently, a 300-nm-thick aluminum (Al) film was deposited on the SiO_2_/Si substrate by thermal evaporation. Next, the anodizing of the Al film was carried out in 10 wt.% phosphoric acid with a 60-V bias. Subsequently, the pores were widened in 5 wt.% phosphoric acid. Then, inductively coupled plasma etching was performed to excavate the barrier layer at the bottom of the AAO pores and the SiO_2_ layer as well as to pattern the surface of the Si substrate under a Cl_2_/BCl_3_ plasma. This step was followed by the removal of the AAO mask and the SiO_2_ layer. Subsequently, a layer of gold (Au) film was deposited onto the patterned Si (100) substrate using an ion-sputter coater, which formed a mesh-like Au film on the Si substrate. Finally, the ordered arrays of vertically aligned SiNWs were obtained by immersing the Au mesh-covered silicon substrate into an etching solution of hydrofluoric acid (HF, 4.4 M)/hydrogen peroxide (H_2_O_2_, 0.4 M) for the metal-assisted chemical etching. The morphology of the samples was characterized by scanning electron microscopy (SEM; Hitachi S-4800, Hitachi Ltd., Chiyoda-ku, Japan).

## Results and discussion

### Structure of the patterned Si substrate

**Figure 2:**
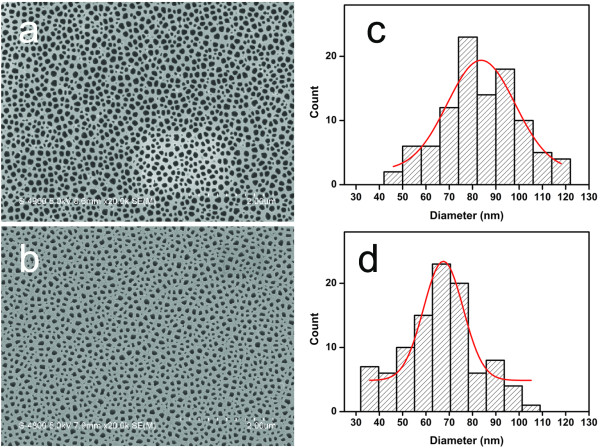
**SEM image and diameter distribution of the patterned silicon surface and the Au mesh.** (**a**) SEM image and (**c**) diameter distribution of the patterned silicon surface after the removal of the AAO mask and SiO_2_ layer. (**b**) SEM image and (**d**) diameter distribution of the Au mesh after the deposition of the Au film on the patterned silicon surface. The bars in (c) and (d) represent the measured statistical data, and the line is a Gaussian fitting.

The SEM image and the statistical diameter distribution of the patterned silicon (100) surface after the removal of the AAO mask and SiO_2_ layer (corresponding to Figure 1d) are shown in Figure 2a,c. The average hole diameter and hole density were estimated to be 84 nm ± 19%, and 5.6 × 10^9^/cm^2^, respectively. The large standard deviation of the hole diameter distribution originates primarily from the dispersity of the AAO pore size, which might be due to the very small thickness of the Al film and the unoptimized anodizing conditions compared with the typical anodization process [18,19]. Figure 2b shows the SEM image of the Au mesh film obtained after depositing the Au film on the patterned silicon (100) surface by ion sputtering (corresponding to Figure 1e). Due to the closure effect [13], the average apertures of the Au mesh decrease with increased thickness of the Au film. After depositing a 45-nm-thick Au film, the average hole diameter decreases to 65 nm ± 15%, as shown in Figure 2d.

**Figure 3:**
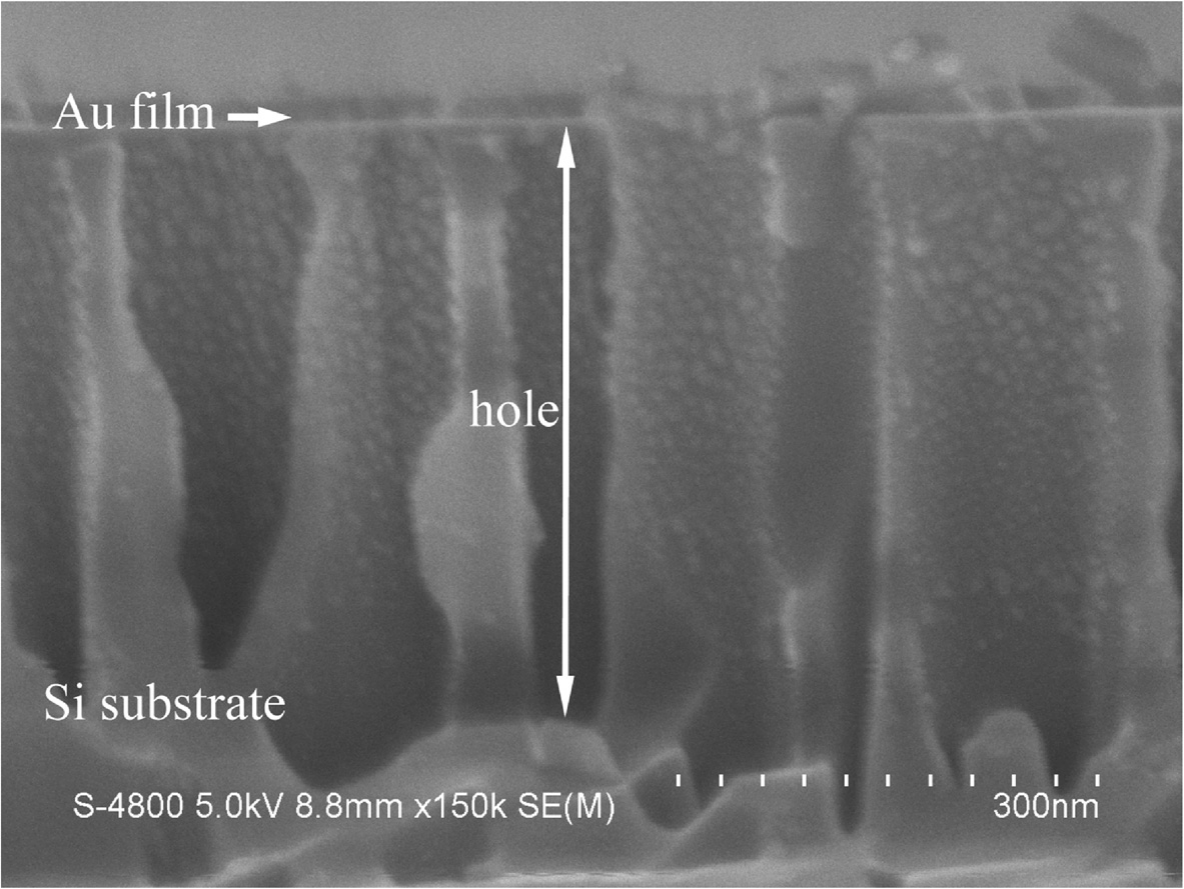
Cross-sectional SEM image of the patterned Si substrate covered with Au film.

The sputtering process resulted in a uniform deposition of the Au on the top surface of the patterned silicon, partially coating on the upper side walls, but not on the bottom of the holes, as shown in Figure 3, which can be primarily attributed to the large depth-width ratio of the holes (approximately 5.6), considering the poor step coverage and the undemanding deposition conditions of ion sputtering.

### Structure of the SiNW arrays

**Figure 4:**
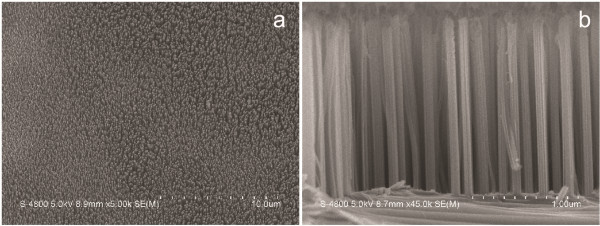
**Plan-view (a) and cross-sectional (b) SEM images of the large-area, vertically aligned SiNW arrays.** For the SEM observation, the sample was tilted by 15°.

The resulting large-area, vertically aligned SiNW array is shown in Figure 4a. Upon close examination, Au can be clearly observed at the interfacial region between the SiNWs and the substrates, while no Au particle is found on the top of each SiNW. This result is consistent with the observation that Au is not deposited at the bottom of the holes (see Figure 3). Figure 4b shows that the SiNW exhibits a uniform diameter along the height direction, indicating that Au is inert against oxidative dissolution in the etching solution and is superior to the Ag catalyst which resulted in the tapered morphologies of the SiNWs with larger diameters at the bottom part due to the dissolution-induced gradual increase of the hole sizes of the Ag mesh during etching [12,13].

### Effect of Au mesh thickness on the etching rate

**Figure 5:**
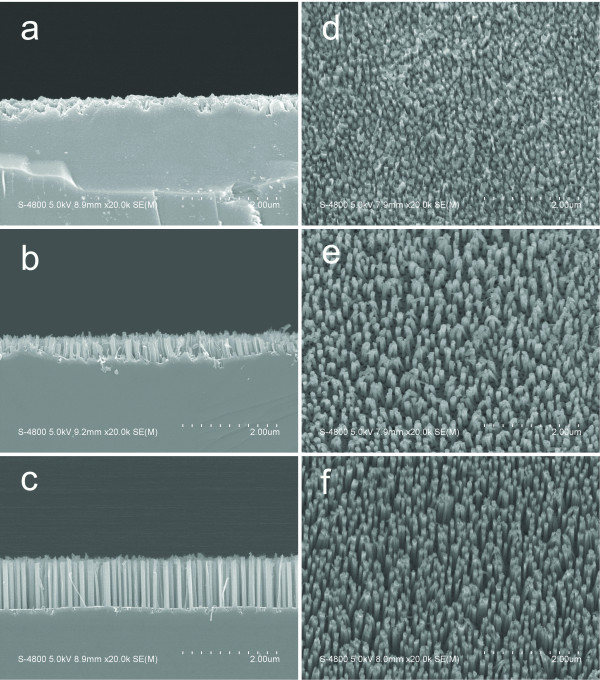
**SEM images of the SiNW arrays catalyzed using the Au mesh with different thickness.** Cross-sectional (**a**, **b**, **c**) and the corresponding plan-view (**d**, **e**, **f**) SEM images of the vertically aligned SiNW arrays catalyzed using the Au mesh with thicknesses of 15, 30, and 45 nm, respectively, for 10 min at 22°C. For the SEM observation, the samples were tilted by 15°.

**Figure 6:**
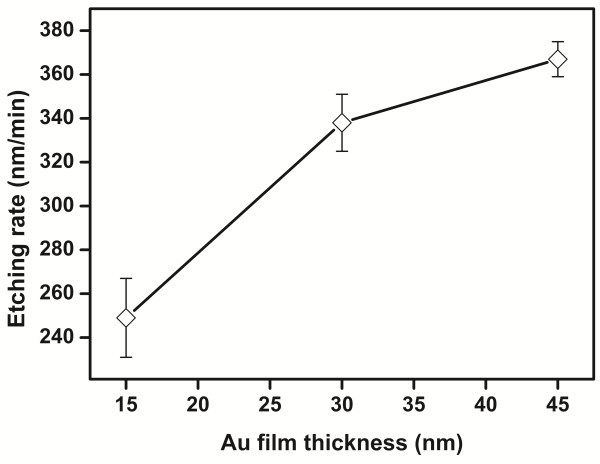
Relationship of the thickness of the Au film and the etching rate of the Si substrate.

The Au films with thicknesses of 15, 30, and 45 nm were deposited on the same patterned Si substrate and then subjected to metal-assisted chemical etching for 10 min at 22°C. Interestingly, the height of the SiNWs catalyzed using a thick Au mesh was much larger compared with that catalyzed using a thin one (see Figure 5). The average heights of the resulting SiNWs are 220, 458, and 1,076 nm, respectively. Clearly, the disparity in the height of the SiNWs can be attributed to the different etching rates of the Si catalyzed using the Au meshes with different thicknesses. In the present study, prior to the formation of the SiNWs, the approximately 470-nm-deep silicon wall (see Figure 3) surrounding the hole must be firstly etched away. The etching rate of the silicon wall may be not the same as that of the silicon substrate under this porous layer because of the different circumstance. To achieve the etching rate of the silicon substrate, i.e., the formation rate of the SiNWs, the samples were etched for a longer duration while keeping the other conditions the same as in the previously mentioned case wherein the etching was carried out for 10 min. Supposing a linear relationship between the SiNW height and the etching duration [14], the etching rate can be calculated by comparing the heights of the SiNWs with those etched for 10 min; the results are shown in Figure 6. Clearly, a high etching rate (>250 nm/min) was obtained in the present conditions, and the etching rate increases with increasing thickness of the Au film. The etching was also performed at a solution temperature of 28°C. The same trend was observed with a higher etching rate of over 400 nm/min.

### Mechanism for difference in the etching rate

**Figure 7:**
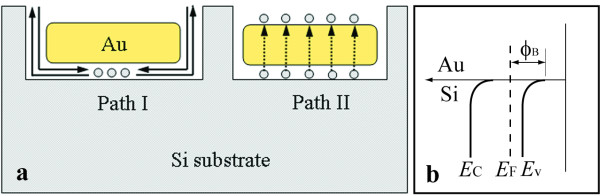
**Schematic of the reagent and by-product diffusion paths and diagram of the Au/Si Schottky contact.** (**a**) Schematic of two possible diffusion paths of the reagent and by-product during the metal-assisted chemical etching process. (**b**) Energy band diagram of the Au/Si Schottky contact; *Φ*_B_ is the barrier height for the electronic holes injected from the Au into the Si.

The result above is the first to cite the difference in the silicon etching rate induced using a Au film with different thicknesses. The exact mechanism is not clear at the moment. The etching rate might be controlled by the mass transfer process of the reagent and the by-product [13,14]. A short diffusion path facilitating the rapid mass transfer of the reagent and the by-product is expected to result in a high etching rate. Figure 7a schematically illustrates the possible diffusion paths of the reagent and the by-product in the Si etching process. In path I, the reagent and the by-product diffuse along the interface between the Au film and the Si, which signifies that the etching rate decreases with the increase in the lateral size of the Au catalyst because of the long lateral diffusion distance. In path II, the Si atoms underneath the Au are dissolved in the Au and then diffuse through the Au film to the Au/solution interface where the silicon atoms are oxidized and etched away [14,20]. On one hand, if the etching rate is dominated by the mass transfer through path I during the chemical etching, a thick Au mesh should lead to a low etching rate because of the increasing lateral size of the Au catalyst caused by the shrinking of the holes induced by the closure effect (see Figure 2). Meanwhile, for a sample etched using a Au mesh with a certain thickness, the irregular interpore distance in the Au mesh, that is, the various lateral diffusion distances for the reagent and the by-product, should result in a fluctuation of height in the SiNW array. On the other hand, if the dominant mass transfer path is path II, a low etching rate for the thick Au mesh can also be inferred because of the large diffusion distance along the vertical direction. However, the present study shows that the thick Au mesh induces a high etching rate, and the SiNWs in the same sample have almost identical heights, especially for the SiNW arrays with large heights (see Figures 4b and 5d). The observations contradict the predictions for both models. Therefore, the mass transfer process can be concluded as a non-dominant factor with regard to the different etching rates.

The difference in the etching rates is naturally attributed to the charge transfer process. An oxidation-reduction reaction is well accepted to occur during the etching of the Si in a solution containing HF and H_2_O_2_[14,20]. The H_2_O_2_ is preferentially reduced at the noble metal surface, thereby generating electronic holes h^+^ according to reaction 1 (cathode reaction) [20]:

(1) 2H++HO22→2H2O+2h+

At the anode, the generated electronic holes are injected into the Si substrate in contact with the metal, leading to the oxidation and then to the dissolution of the Si underneath the metal according to reaction 2 [20]:

(2) $$\mathrm{Si}+6H^{+}+6F^{-}+4h^{+}\to \mathrm{Si}F_{6}^{2-}+6H^{+}$$

The charge transfer between the Si and the Au would be heavily affected by the Au/Si Schottky barrier height (see Figure 7b). It has been reported that the size of the metal has an important effect on the surface band bending of Si [13,14]. The Schottky barrier height of the semiconductor/metal contact is said to increase with the decrease of the feature size of the metal [13,21,22]. Based on the results and discussions above, the thickness of the Au mesh, and not the lateral size, can be suggested as the factor that determines the Au/Si Schottky barrier height, considering the continuous property of the Au mesh. The barrier height *Φ*_B_ decreases with the increase of the thickness of the Au mesh. Therefore, electronic holes can be easily injected from the thick Au mesh into the Si substrate underneath the Au because of the reduced barrier height compared with that of the thin Au mesh, thus, resulting in a high etching rate.

According to the model of charge transfer through the Schottky barrier, ideally, the Fermi energy level determined by the doping type and doping level of the Si substrate and the work function of the noble metal will determine the surface band bending of Si, thus affecting the hole injection from the noble metal to the Si and, furthermore, affecting the etching rate. In fact, n-doped Si was found to be etched faster than p-doped Si [17,23], and the etching rate decreases with increasing dopant concentration for both n- and p-doped Si [11,17,24]. Meanwhile, Li et al. reported that the etching rate showed only small variation for a Au-coated p^+^, p^−^, and n^+^ Si substrate and a Pt-coated Si was etched faster compared with a Au-coated Si [25]. Obviously, abovementioned experiment results cannot be accounted for only by the charge transfer through an ideal Schottky barrier. A rigorous model should consider the full process of charge transfer including the generation of holes, diffusion in the metal, going through the Schottky barrier, as well as diffusion in the Si substrate, which involved the catalytic activity of the noble metal for oxidant (affecting the generation rate of holes), the surface state of Si, the diffusion of holes from the etching front to off-metal areas or to the sidewall of the formed structure (especially in a heavily doped Si, resulting in the formation of a porous structure), etc. [14,17]. However, this has not been done so far, and it needs to be further explored.

Metal-assisted chemical etching of Si allows fabricating large-area SiNWs with predetermined doping type and doping level. By utilizing the AAO template, the diameter, spacing, and areal density of nanowires can be further controlled through optimizing the anodizing conditions. Moreover, the SiNWs fabricated by this method are well-discrete and vertically aligned, which is critical for subsequent coating of other layers in device fabrication. Therefore, this technique is very promising for device fabrication based on SiNW array, for instance, SiNW radial p-n junction solar cells [6].

## Conclusions

In conclusion, combining the AAO template and the metal-assisted chemical etching process results in large-area, vertically aligned SiNWs with a uniform diameter along the height direction. The thickness of the Au film was found to affect the etching rate of Si, which might be caused primarily by the charge transfer process. A thick Au mesh that comes in contact with Si reduces the Au/Si Schottky barrier height, which facilitates the injection of electronic holes from the Au mesh into the Si, thereby resulting in a high etching rate of Si. This method provides a simple and low-cost approach to the control of the doping type, doping level, diameter, spacing, areal density of SiNW arrays, etc. Well-discrete and vertically aligned SiNW array fabricated by this method is very promising for device applications based on SiNW arrays.

## Competing interests

The authors declare that they have no competing interests.

## Authors' contributions

ZZ carried out the preparation and main characterization of the SiNWs and drafted the manuscript. GC participated in its design and coordination. YS participated in the design of the study. YL participated in the data analysis and English description modification. GJ participated in the mechanism analysis of different etching rates of SiNWs. All authors read and approved the final manuscript.
